# Proteome Profiling Identifies Serum Biomarkers in Rheumatoid Arthritis

**DOI:** 10.3389/fimmu.2022.865425

**Published:** 2022-05-06

**Authors:** Congqi Hu, Zhao Dai, Jia Xu, Lianyu Zhao, Yanping Xu, Meilin Li, Jiahui Yu, Lu Zhang, Hui Deng, Lijuan Liu, Mingying Zhang, Jiarong Huang, Linping Wu, Guangxing Chen

**Affiliations:** ^1^ Department of Rheumatology, First Affiliated Hospital of Guangzhou University of Chinese Medicine, Guangzhou, China; ^2^ First Clinical Medical School, Guangzhou University of Chinese Medicine, Guangzhou, China; ^3^ Baiyun Hospital of the First Affiliated Hospital of Guangzhou University of Chinese Medicine, Guangzhou, China; ^4^ Center for Chemical Biology and Drug Discovery, Guangzhou Institute of Biomedicine and Health, Chinese Academy of Sciences (CAS), Guangzhou, China

**Keywords:** rheumatoid arthritis, biomarker, proteomics, ORM1, serum

## Abstract

Rheumatoid arthritis (RA) causes serious disability and productivity loss, and there is an urgent need for appropriate biomarkers for diagnosis, treatment assessment, and prognosis evaluation. To identify serum markers of RA, we performed mass spectrometry (MS)-based proteomics, and we obtained 24 important markers in normal and RA patient samples using a random forest machine learning model and 11 protein–protein interaction (PPI) network topological analysis methods. Markers were reanalyzed using additional proteomics datasets, immune infiltration status, tissue specificity, subcellular localization, correlation analysis with disease activity-based diagnostic indications, and diagnostic receiver-operating characteristic analysis. We discovered that ORM1 in serum is significantly differentially expressed in normal and RA patient samples, which is positively correlated with disease activity, and is closely related to CD56dim natural killer cell, effector memory CD8^+^T cell, and natural killer cell in the pathological mechanism, which can be better utilized for future research on RA. This study supplies a comprehensive strategy for discovering potential serum biomarkers of RA and provides a different perspective for comprehending the pathological mechanism of RA, identifying potential therapeutic targets, and disease management.

## Introduction

Rheumatoid arthritis (RA) is an autoimmune disease characterized by chronic erosive arthritis, which affects 1% of the world’s population and usually occurs in middle-aged and elderly women (1). The clinical manifestations are symmetrical and persistent polyarticular swelling and pain and can also involve extra-articular tissues, such as skin and mucosal lesions, cardiovascular diseases, and lung diseases, which seriously affect work and daily life (2, 3).

Current common laboratory indicators are rheumatoid factor (RF), anti-cyclic citrullinated peptide antibody (ACPA), erythrocyte sedimentation rate (ESR), and C-reactive protein (CRP) (4). However, the RF-positive rate and ACPA specificity of RA are only 60%–70% and 60%–75%, respectively, suggesting low diagnostic efficacy (5, 6). Furthermore, the RF-positive rate and ACPA specificity are difficult to distinguish between high and low disease activity. ESR or CRP can reflect the inflammatory activity of the disease but is not a characteristic diagnosis of RA (7). The situation largely limits the timely and efficient treatment and analysis of patients, Thus, the diagnosis of the disease can still be much improved. Biomarkers specifically and powerfully related to RA disease have been increasing; it can be helpful especially in patients who are seronegative for RF and ACPA.

It is known that proteins have an important role in RA pathogenesis with the formation of a new epitope; compared with other techniques such as transcription, proteomics can study the posttranscriptional modification of proteins and their interactions in cells and tissues (8). Previous proteomic studies according to quantitative analyses using clinical serum samples between RA patients and healthy controls identified a lot of differentially expressed proteins. Cheng et al. set up protein profiles of serum through high-resolution mass spectrometry using the Orbitrap Q Exactive mass spectrometer and identified 18 overexpressed proteins, among which FCN-2 was found to be elevated in RA and correlated with disease activity (9). Sora et al. using a nano-LC-MS/MS-based proteomics approach speculated that SAA might be a biomarker of AA amyloidosis in rheumatoid arthritis; gelsolin and VDBP might be potent biomarker candidates for the early diagnosis of RA (10). Lee et al. suggested that protein S100-A9 is involved in joint destruction by matrix metalloproteinases and the pro-inflammatory response, showing potential for use as a diagnostic biomarker that reflects the mechanism of inflammation in RA (10). However, these studies only aimed at identifying the protein difference between normal and RA patients, rarely combined with disease activity, and noticed differences in protein expression after drug intervention.

**Figure 1:**
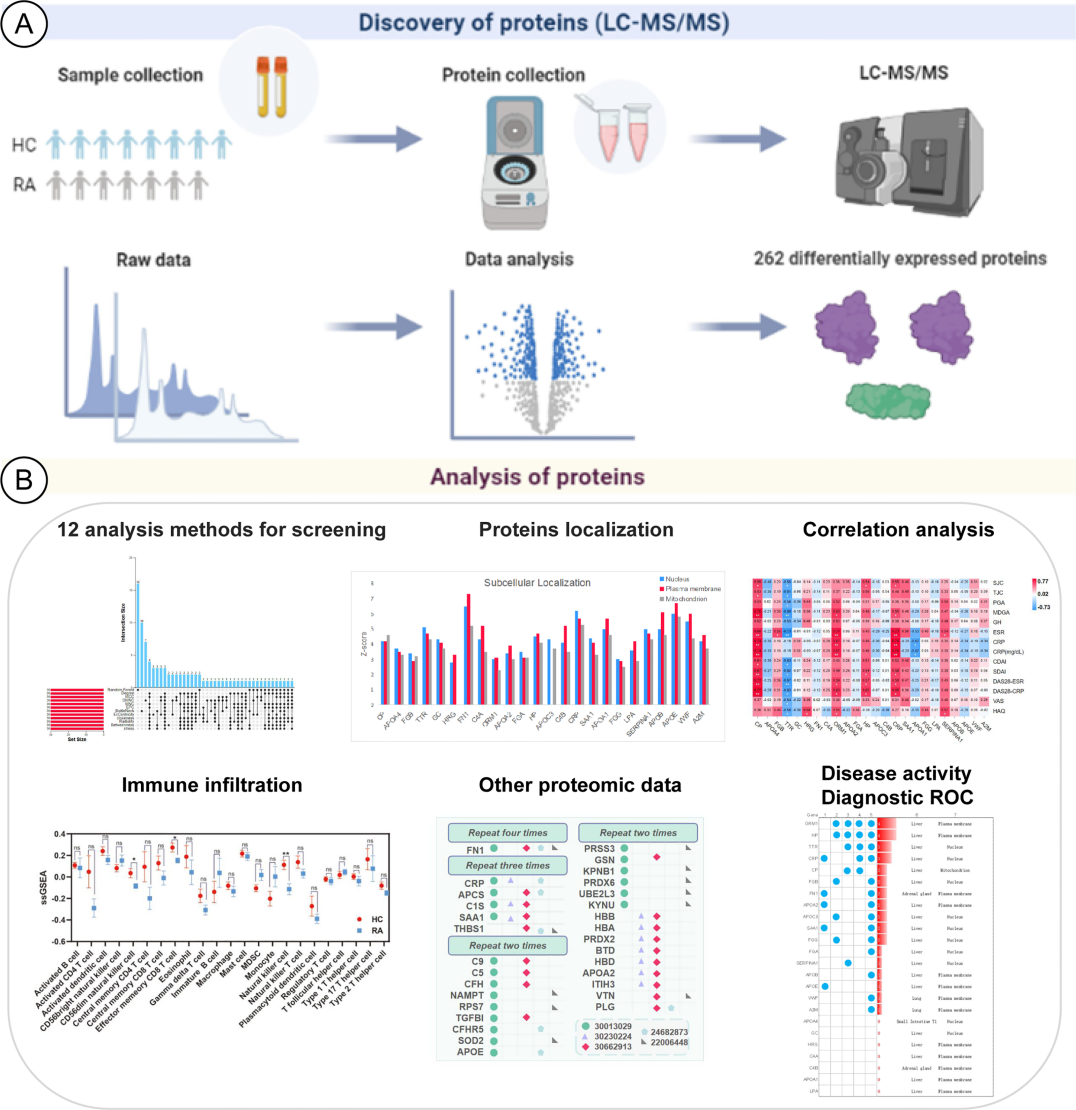
Schematic diagram about the analysis of RA potential proteins based on LC-MS proteomics. **(A)** Identification of differentially expressed proteins. **(B)** Multiple-analysis methods to assess the importance of differentially expressed proteins.

In this study, our comprehensive analysis of serum proteins in RA from multiple perspectives is shown (**Figure 1**). We obtained differential proteins based on serum proteomics by LC-MS/MS, and we referred to the results of other proteomics datasets. Analysis of immune infiltration, tissue and subcellular localization, and the correlation between differential proteins and RA laboratory diagnostic indicators and diagnostic receiver-operating characteristic analysis from the perspective of disease activity were performed. These results provide a worthy reference for future proteomic and multi-angle analysis studies on RA serum protein biomarkers.

## Methods and Materials

### Patients

Human serum specimens were collected from the First Affiliated Hospital of Guangzhou University of Chinese Medicine (Guangzhou, China). The study was approved by the Chinese Clinical Trial Registry (ChiCTR), and the registration number is ChiCTR2100043294. This study was approved by the local research ethics committee; the approval document number of the ethics committee is NO. JY[2020]264. All subjects in this study were asked for consent. Human serum samples included 8 healthy control and 7 patients at different time points of 0 and 6 after administration. All samples were frozen and stored at −80°C in aliquots of polyethylene tubes until use.

### Protein Preparation

Bicinchoninic acid (BCA) assay was used for protein quantification, taking 500 μg of protein and using High Select™ Top14 Abundant Protein Depletion Resin to remove high-abundance proteins. After adding the supernatant obtained in the previous step to dithiothreitol (DTT) at a final concentration of 10 mM, polypeptide samples were prepared using the filter-aided sample preparation (FASP) method from previous literatures (11, 12). In brief, proteins were added onto a 30-kDa cutoff filter and centrifuged at 11,000 rpm at 20°C for 15 min. 50 mM iodoacetamide (IAA) in urea buffer was used to alkylate proteins at 20°C for 15 min. After a few washes with urea and 50 mM ABC buffer, 100 ng of trypsin in 50 mM ABC buffer was used to digest proteins in a wet chamber overnight at 37°C. Peptides were extracted with 50 mM ABC buffer and acidified by trifluoroacetic acid (TFA). After the FASP protocol as described above, the peptides were desalted and concentrated using C18 stage tips. Each of these samples was measured in a 1-h gradient of LC-MS/MS.

### Protein Quantification and Analysis

Tryptic peptides were separated using a 60-min total data collection for peptide separation, with the Easy-nLC 1200 system connected online to an Orbitrap Eclipse mass spectrometer equipped with FAIMS Pro (Thermo). Scans were collected in data-dependent top-speed mode with dynamic exclusion at 90 s. Raw data were analyzed using MaxQuant version 1.6.17.0 search against the Human Fasta database, with label-free quantification and match-between-run functions enabled. The identification of proteins that were differentially expressed between patients with RA and HCs was conducted based on the empirical Bayes method using the limma R package. The *p*-values were obtained by using the t-test, and the *p*-values of comparisons were adjusted for multiple comparisons to preserve an error rate of 5%. To identify the significant pair(s), we used multiple comparisons. The Benjamini–Hochberg method was used for the multiple comparisons.

### Identification of Differentially Expressed Proteins

#### Key Protein Screening

Machine learning was combined with PPI network analysis to screen differential proteins. The random forest classifier (RFC) was used to perform feature extraction on protein sample expression data, screen out the top 30 key proteins based on classification features, and use MeanDecreaseGini to plot. 11 topological analysis methods, including Degree, Edge Percolated Component (EPC), Maximum Neighborhood Component (MNC), Density of Maximum Neighborhood Component (DMNC), Maximal Clique Centrality (MCC), and six centralities (Bottleneck, EcCentricity, Closeness, Radiality, Betweenness, and Stress) based on shortest paths were used to measure the true efficiency of nodes in the PPI network to evaluate their importance.

#### Enrichment Analysis

R (version 3.6.3)’s ggplot2 package and clusterProfiler package were used to analyze the enrichment analysis of 24 differential proteins, including KEGG pathway, biological process (BP), cell component (CC), and molecular function (MF).

### Compilation of Other Proteomic Datasets

“rheumatoid arthritis” and “proteomics” were used as keywords, PubMed (https://pubmed.ncbi.nlm.nih.gov/) was used to search, and clinical samples with healthy people were selected as the control group. A total of 5 datasets were collected, including 155 healthy controls and 183 RA patients. The differentially expressed proteins were summarized in each dataset, and candidate proteins with the number of repetitions greater than 2 were selected. An NCBI gene ID was assigned to each protein based on gene symbol, and we kept proteins with the NCBI gene IDs for subsequent analysis.

### Multi-Angle Analysis of Differentially Expressed Proteins

#### Immune Infiltration

The R (version 3.6.3) ssGSEA method was used for immune cell infiltration analysis, the degree of infiltration of 28 immune cells was calculated, and *p* < 0.05 was considered statistically significant. The following 28 types of immune cells were obtained: activated B cells, activated CD4^+^T cells, activated CD8^+^T cells, activated dendritic cells, CD56bright natural killer cells, CD56dim natural killer cells, central memory CD4^+^T cells, central memory CD8^+^T cells, effector memory CD4^+^T cells, effector memory CD8^+^T cells, eosinophils, follicular helper T cells, gamma delta T cells, immature B cells, immature dendritic cells, macrophages, mast cells, memory B cells, monocytes, myeloid-derived suppressor cells, natural killer cells, natural killer T cells, neutrophils, plasmacytoid dendritic cells, regulatory T cells, type 1 T helper cells, type 17 T helper cells, and type 2 T helper cells. We used the Spearman correlation analysis method to compare the obtained immune cells and correlation analysis of key proteins to further discover RA disease proteins closely related to immune infiltration.

#### Tissue Specificity and Subcellular Localization

Tissue specificity data were obtained from GTEx (https://gtexportal.org/). GTEx is a tissue-specific database of gene expression and regulation derived from simultaneous transcriptome sequencing and genetic analysis of samples from multiple human tissues and organs.

Subcellular localization was performed through COMPARTMENTS (https://compartments.jensenlab.org/). COMPARTMENTS is a powerful website for predicting the subcellular localization of proteins, helping us to better understand candidate proteins.

### Analyze Differential Proteins in Combination With Disease Activity

#### Diagnostic Correlation Analysis

R (version 3.6.3) was used for statistical analysis and visualization, the ggplot2 package (version 3.3.3) was used for visualization, and the statistical method used was Spearman. A heatmap was used to display the pairwise correlation (spearman correlation) of all elements between the two tables (protein expression table and diagnostic index change table). Statistical significance was marked on the heatmap.

#### Diagnostic ROC Analysis

We used the pROC package for diagnostic ROC analysis and used the ggplot2 package for visualization. The value of the area under the curve (AUC) is between 0.5 and 1. The closer the AUC is to 1, the better the diagnostic effect. The AUC has a low accuracy when it is 0.5–0.7, it has a certain accuracy when it is 0.7–0.9, and it has a high accuracy when it is above 0.9; key proteins were further screened based on an AUC greater than 0.7.

#### Statistical Analysis and Network Visualization

The statistical analysis in this study was performed by the differential enrichment test of R platforms (version3.6.3, http://www.r-project.org/). Data visualization was generated by the clusterProfiler package (version3.14.3) of R and Microsoft Office 2019.

## Results

### Identification of Proteins by LC-MS/MS

Raw counts of proteomics data and corresponding clinical information from healthy control and RA patients were obtained from the First Affiliated Hospital of Guangzhou University of Chinese Medicine (Guangzhou, China). The differential enrichment test of R software was used to study the differential expression of proteins. Test-diff was used to perform a differential enrichment test based on protein-wise linear models and empirical Bayes statistics using limma. False discovery rates were estimated using the fdrtool package (version 1.2.17). *p*-value < 0.05 was defined as the threshold for the screening of differential expression. A total of 2,051 proteins and peptides were identified by LC-MS/MS. There were a total of 262 candidate proteins that are statistically significant between healthy control cases and RA patients, and a total of 93 candidate proteins during the active and inactive phases of the disease.

### Identify Candidates Proteins for RA

#### Key Protein Screening

**Figure 2:**
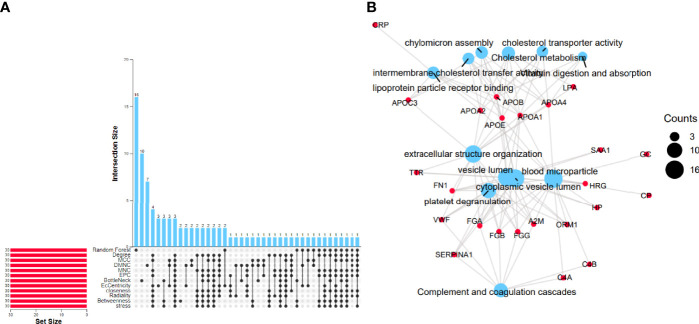
Identification of differentially expressed proteins. **(A)** Random forest machine learning method combined with the PPI network importance analysis algorithm to screen differential proteins. **(B)** Enrichment results of 24 differential proteins. The blue size represents the number of counts corresponding to the result.

Analyzing 262 candidate proteins, according to the RFC machine learning method and 11 topological analysis methods (**Figure 2A**), the top 30 key proteins under each method are obtained. Proteins present in more than 8 of the 12 analytical methods were defined as key proteins for follow-up research, and 24 proteins obtained were CP, APOA4, FGB, TTR, GC, HRG, FN1, C4A, ORM1, APOA2, FGA, HP, APOC3, C4B, CRP, SAA1, APOA1, FGG, LPA, SERPINA1, APOB, APOE, VWF, and A2M.

#### Enrichment Analysis

To further confirm the underlying function of potential targets, we use Fisher’s exact test to calculate the enrichment significance of each term in KEGG, biological process (BP), cell composition (CC), and molecular function (MF) and arrange them in ascending order of *p*-value. **Figure 2B** visually displays the relationship between the proteins and KEGG, BP, CC, and MF.

24 proteins overlapping the reference datasets were analyzed. The first 3 KEGG pathways with the smallest p-value were “Complement and coagulation cascades (TermID: hsa04610, *p*-value<0.001); Cholesterol metabolism (TermID: hsa04979, *p*-value<0.001); Vitamin digestion and absorption (TermID: hsa04977, *p*-value<0.001).” The first 3 BPs with the smallest p-value were extracellular structure organization “(TermID: GO:0043062, *p*-value<0.001); platelet degranulation (TermID: GO:0002576, *p*-value<0.001); chylomicron assembly (TermID: GO:0034378, *p*-value<0.001).” The first 3 CCs with the smallest p-value were “blood microparticle (TermID: GO:0072562, *p*-value<0.001); cytoplasmic vesicle lumen (TermID: GO:0060205, *p*-value<0.001); vesicle lumen (TermID: GO:0031983, *p*-value<0.001).” The first 3 MFs with the smallest p-value were “lipoprotein particle receptor binding (TermID: GO:0070325, *p*-value<0.001); cholesterol transporter activity (TermID: GO:0017127, *p*-value<0.001); intermembrane cholesterol transfer activity (TermID: GO:0120020, *p*-value<0.001)”.

#### Identify Proteins from Other Proteomics Datasets

In order to systematically investigate the biomarkers related to the pathogenesis of RA, we conducted a comprehensive analysis of 5 independent RA proteomic datasets. These datasets cover 5 ultra-deep reference datasets from FLS and serum. The five in-depth reference datasets were as shown in **Table 1**. We collected 450 DEPs from 183 RA patients and 155 healthy control cases and established a relatively comprehensive RA proteomic dataset.

**Table 1:** Literature summary of differential proteins.

Official Full Name	Symbol	Total articles	RA related articles	PMID	Level Change
Orosomucoid 1	**ORM1**	3,701	5	26915672	Up
Haptoglobin	**HP**	96,237	996	4029960	Up
Transthyretin	**TTR**	13,127	61	30308029	Down
C-Reactive Protein	**CRP**	108,764	6,602	33385862	Up
Ceruloplasmin	**CP**	139,242	649	26001728	Up
Fibrinogen Beta Chain	**FGB**	1,920	15	26059223	Up
Fibronectin 1	**FN1**	25,482	14	34764682	Down
Apolipoprotein A2	**APOA2**	3,263	3	12027302	Down
Apolipoprotein C3	**APOC3**	1,230	2	31966382	Down
Serum Amyloid A1	**SAA1**	986	52	17985847	Up
Fibrinogen Gamma Chain	**FGG**	2,003	11	32408093	Up
Fibrinogen Alpha Chain	**FGA**	3,606	10	22267327	Up
Serpin Family A Member 1	**SERPINA1**	909	9	34712268	Up
Apolipoprotein B	**APOB**	20,164	93	29997113	Down
Apolipoprotein E	**APOE**	33,596	53	32253242	Up
Von Willebrand Factor	**VWF**	24,468	129	25973092	Up
Alpha-2-Macroglobulin	**A2M**	5,871	113	10343526	Up
Apolipoprotein A4	**APOA4**	1,212	1	2547867	Up
GC Vitamin D Binding Protein	**GC**	158,417	727	3874814	Down
Histidine Rich Glycoprotein	**HRG**	1,662	6	29246875	Up
Complement C4A	**C4A**	1,813	44	22076784	Down
Complement C4B	**C4B**	3,242	79	22076784	Up
Apolipoprotein A1	**APOA1**	15,810	60	31694752	Down
Lipoprotein A	**LPA**	16,481	114	19369465	Up

**Figure 3:**
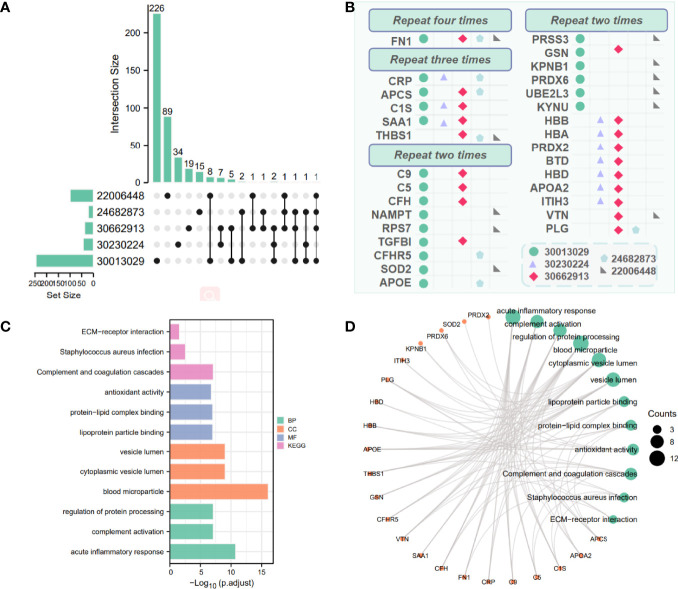
Combine other proteomics datasets to analyze differential proteins. **(A)** Crossover situation in 5 proteomics datasets. **(B)** Visually display differential proteins co-expressed (greater than 2) in multiple proteomics datasets. Different shapes and colors represent different datasets. **(C, D)** Enrichment results of 30 differential proteins in 5 proteomics datasets. The green size represents the number of counts corresponding to the result.

The DEPs of 5 reference datasets were statistically sorted, and the number of overlaps greater than 2 was used as the screening condition; 30 proteins were obtained as potential biomarker proteins. We used the upset diagram (**Figure 3A**) to visually show the overlap of each dataset. **Figure 3B** shows the 30 overlapping proteins and their respective datasets.

To further confirm the underlying function of potential targets, we used Fisher’s exact test to calculate the enrichment significance of each term in KEGG, biological process (BP), cell composition (CC), and molecular function (MF) and arrange them in ascending order of *p*-value. The bar chart in **Figures 3C, D** visually displays Log10 (*p*-adjust), and **Figure 3C, D** visually display the relationship between the proteins and KEGG, BP, CC, and MF.

The 30 proteins overlapping the reference datasets were analyzed. The first 3 KEGG pathways with the smallest *p*-value were “Complement and coagulation cascades (TermID: hsa04610, *p*-value<0.001); Staphylococcus aureus infection (TermID: hsa05150, *p*-value:0.000); ECM-receptor interaction (TermID: hsa04512, *p*-value:0.001).” The first 3 BPs with the smallest *p*-value were “acute inflammatory response (TermID: GO:0002526, *p*-value<0.001); complement activation (TermID: GO:0006956, *p*-value<0.001); regulation of protein processing (TermID: GO:0070613, *p*-value<0.001).” The first 3 CCs with the smallest p-value were “blood microparticle (TermID: GO:0072562, *p*-value<0.001); cytoplasmic vesicle lumen (TermID: GO:0060205, *p*-value<0.001); vesicle lumen (TermID: GO:0031983, *p*-value<0.001)”. The first 3 MF with the smallest p-value were “lipoprotein particle binding (TermID: GO:0071813, *p*-value:0.000); protein-lipid complex binding (TermID: GO:0071814, *p*-value:0.000); antioxidant activity (TermID: GO:0016209, *p*-value:0.000)”.

### Multi-Angle Analysis of Candidate Proteins

#### Immune Infiltration

**Figure 4:**
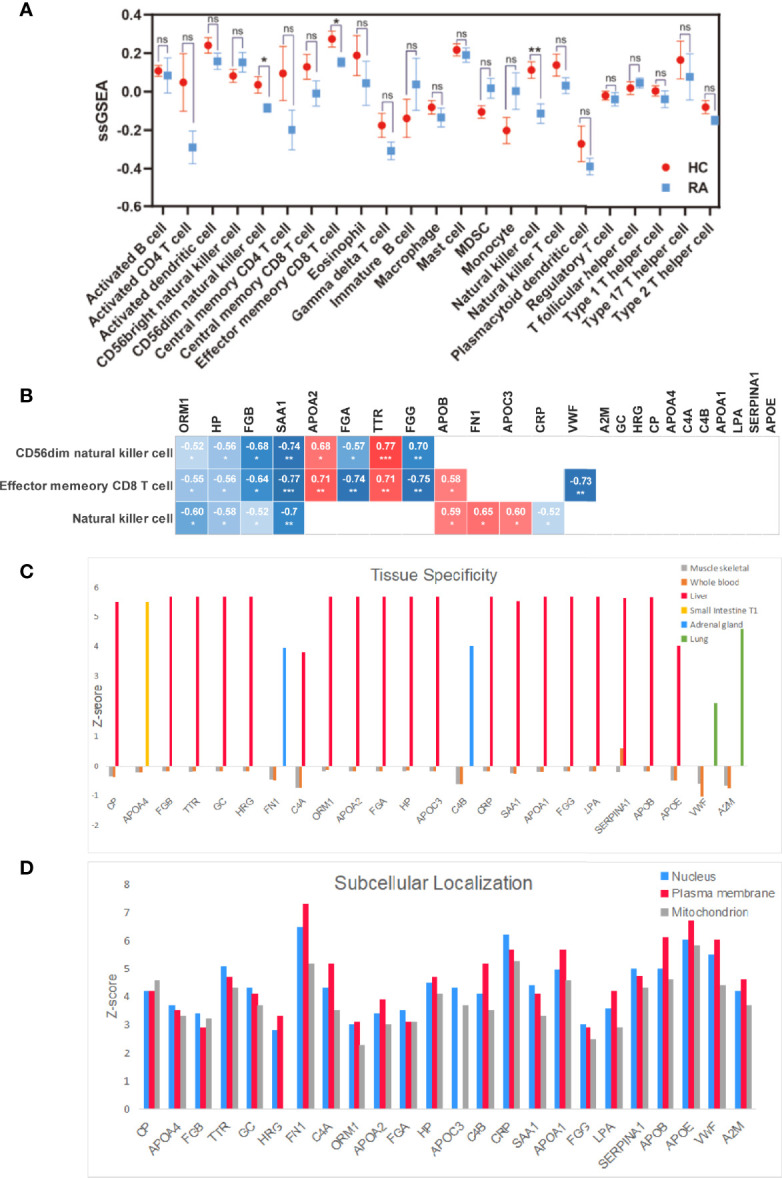
Multi-angle analysis of 24 differentially expressed proteins. **(A)** Immune infiltration analysis protein expression in 23 immune cells in healthy controls and RA patient. **(B)** Correlation analysis of 24 proteins with statistically significant immune cells. **(C)** Tissue specificity of 24 differential proteins. **(D)** Subcellular localization of 24 differential proteins. *p < 0.05, **p < 0.01, ***p < 0.001. ns, no significance.

We found that the degree of immune cell infiltration in RA patients is generally lower than that in normal people. We analyzed 28 types of immune cells and obtained data on 23 types of immune cells, which are activated B cell, activated CD4^+^T cell, activated dendritic cell, CD56bright natural killer cell, CD56dim natural killer cell, central memory CD4^+^T cell, central memory CD8^+^T cell, effector memory CD8^+^T cell, eosinophil, gamma delta T cell, immature B cell, macrophage, mast cell, myeloid-derived suppressor cell, monocyte, natural killer cell, natural killer T cell, plasmacytoid dendritic cell, regulatory T cell, follicular helper cell, type 1 T helper cell, type 17 T helper cell, and type 2 T helper cell. As shown in **Figure 4A**, among 23 immune cells, the degree of immune infiltration of CD56dim natural killer cell, effector memory CD8^+^T cell, and natural killer cell was significantly different between HC and RA (*p* < 0.05, *p* < 0.05, *p* < 0.01). Through Spearman correlation analysis, we found that 13 of the 24 differential genes were significantly related to the degree of immune cell infiltration, which are ORM1, HP, TTR, CRP, FGB, FN1, APOA2, APOC3, SAA1, FGG, FGA, APOB, and VWF; we believe that these 13 genes are potential biomarkers for the treatment of RA and related to immune cell infiltration, as shown in **Figure 4B**.

#### Tissue Specificity and Subcellular Localization

As shown in **Figure 4C**, we studied the tissue specificity of 24 key proteins and found that 21 differential proteins are abundant in the liver (VWF, A2M, CP, FGB, TTR, GC, HRG, C4A, ORM1, APOA2, FGA, HP, APOC3, CRP, SAA1, APOA1, FGG, LPA, SERPINA1, APOB, APOE), FN1 and C4B are abundant in the adrenal gland, VWF and A2M are abundant in lung tissue, and APOA4 is the most abundant in small intestine T1, and these proteins are in whole blood; skeletal muscle protein abundance was relatively low.

As shown in **Figure 4D**, we have studied the subcellular localization of 24 key proteins. Here we focus on their expression on the nucleus, plasma membrane, and mitochondrion and found that 10 proteins (APOA4, FGB, TTR, GC, FGA, APOC3, CRP, SAA1, FGG, SERPINA1) are contained in the nucleus and 13 proteins (HRG, FN1, C4A, ORM1, APOA2, HP, C4B, APOA1, LPA, APOB, APOE, VWF, A2M) are contained in plasma membranes. CP is more abundant in the mitochondrion.

### Analyze Candidate Proteins from Disease Activity

#### Diagnostic Correlation Analysis

**Figure 5:**
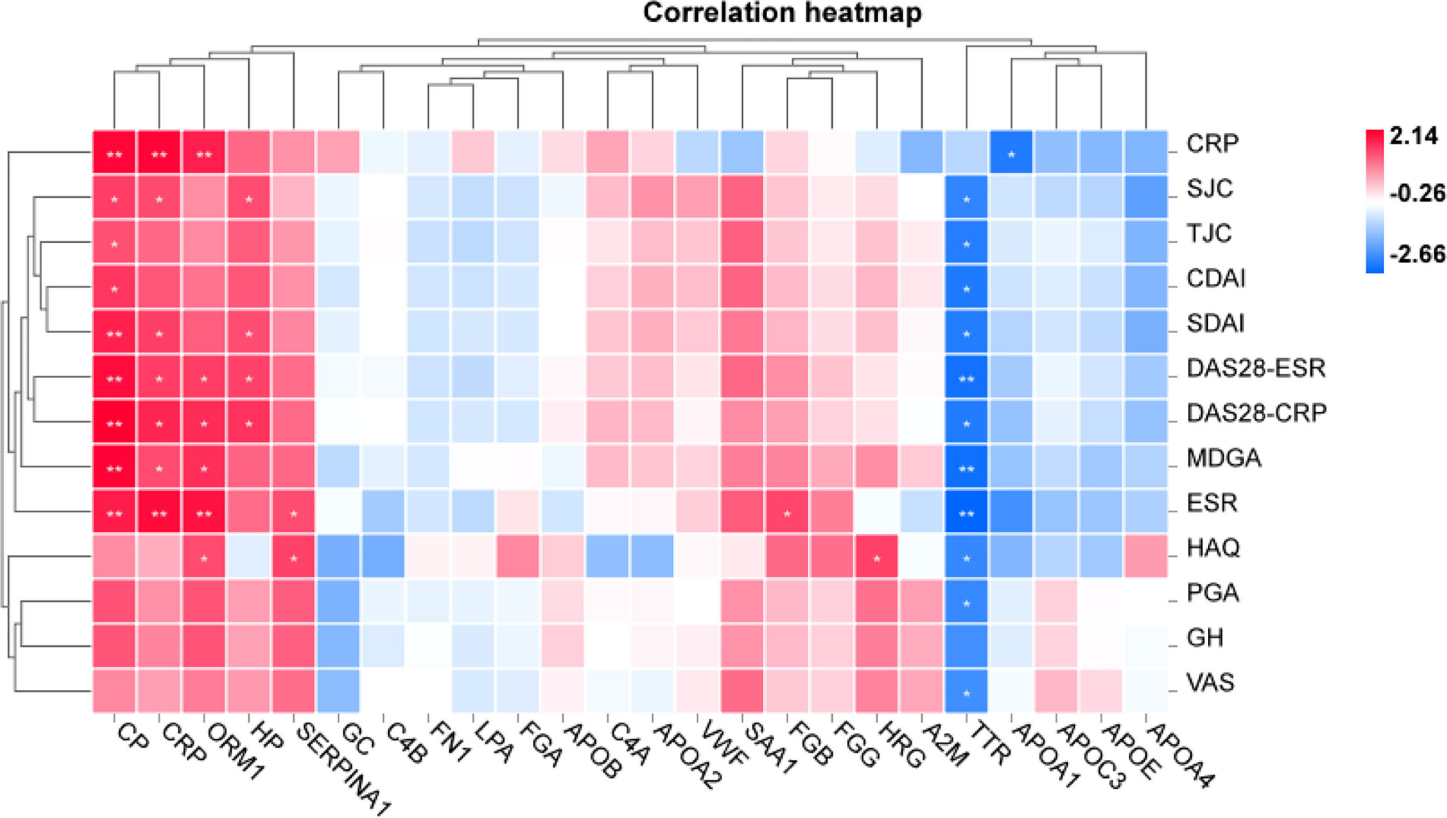
Correlation analysis between RA laboratory diagnostic indicators and disease activity. Positive and negative signs indicate the direction of the correlation. A positive sign indicates a positive correlation (red), and a negative sign indicates a negative correlation (blue). *p < 0.05, **p < 0.01.

**Table 2** shows that changes in diagnostic indicators in patient 13 correlated with disease activity before and after treatment. The judging index of disease activity is DAS28-ESR. It can be seen from **Table 2** that the DAS28-ESR of DAP patients is 5.5, and the DAS28-ESR of DIP after treatment is 2.33 (Wilcoxon test *p*<0.001). After using Spearman correlation analysis between 24 proteins and 13 diagnostic indicators (SJC, TJC, PGA, MDGA, GH, ESR, CRP, CDAI, SDAI, DAS28-ESR, DAS28-CRP, VAS, HAQ), the correlation analysis between CP and 11 diagnostic indicators except CRP and GH was statistically significant, as shown in **Figure 5**. The correlation analysis between CP and 9 diagnostic indicators (CRP, SJC, TJC, CDAI, SDAI, DAS28-ESR, DAS28-CRP, MDGA, ESR) was statistically significant. The correlation analysis between CRP and 7 diagnostic indicators (CRP, SJC, SDAI, DAS28-ESR, DAS28-CRP, MDGA, ESR) was statistically significant. The correlation analysis between ORM1 and 6 diagnostic indicators (CRP, DAS28-ESR, DAS28-CRP, MDGA, ESR, HAQ) was statistically significant. The correlation analysis between HP and 4 diagnostic indicators (SJC, SDAI, DAS28-ESR, DAS28-CRP) was statistically significant. The correlation analysis between SERPINA1 and ESR and HAQ was statistically significant. The correlation analysis between FGB and ESR was statistically significant. The correlation analysis between HRG and HAQ was statistically significant. The correlation analysis between APOA1 and CRP was statistically significant. We chose proteins that were statistically significant in the correlation analysis with more than 3 diagnostic indicators as key proteins. We believe that among the 24 proteins, the five proteins TTR, CP, CRP, ORM1, and HP are more closely related to diagnostic indicators and have the potential to become markers for the diagnosis and treatment of RA.

**Table 2:** Clinical indicator information.

Characteristic	DAP	DIP	*p*	Method
n	7	7		
SJC, n (%)			0.051	Chisq.test
0	0 (0%)	3 (21.4%)		
1	0 (0%)	2 (14.3%)		
3	2 (14.3%)	0 (0%)		
4	2 (14.3%)	0 (0%)		
5	0 (0%)	2 (14.3%)		
7	1 (7.1%)	0 (0%)		
13	1 (7.1%)	0 (0%)		
28	1 (7.1%)	0 (0%)		
TJC, n (%)			0.051	Chisq.test
0	0 (0%)	5 (35.7%)		
1	0 (0%)	1 (7.1%)		
3	0 (0%)	1 (7.1%)		
5	3 (21.4%)	0 (0%)		
6	1 (7.1%)	0 (0%)		
7	1 (7.1%)	0 (0%)		
10	1 (7.1%)	0 (0%)		
28	1 (7.1%)	0 (0%)		
PGA, n (%)			0.051	Chisq.test
0.5	0 (0%)	1 (7.1%)		
1	0 (0%)	3 (21.4%)		
3	0 (0%)	2 (14.3%)		
4	0 (0%)	1 (7.1%)		
5	4 (28.6%)	0 (0%)		
6	1 (7.1%)	0 (0%)		
7	1 (7.1%)	0 (0%)		
9	1 (7.1%)	0 (0%)		
MDGA, n (%)			0.215	Chisq.test
0.5	0 (0%)	1 (7.1%)		
1	0 (0%)	2 (14.3%)		
3	1 (7.1%)	3 (21.4%)		
4	2 (14.3%)	1 (7.1%)		
5	1 (7.1%)	0 (0%)		
6	2 (14.3%)	0 (0%)		
9	1 (7.1%)	0 (0%)		
GH, n (%)			0.101	Chisq.test
5	0 (0%)	1 (7.1%)		
10	0 (0%)	3 (21.4%)		
30	0 (0%)	2 (14.3%)		
40	1 (7.1%)	1 (7.1%)		
50	3 (21.4%)	0 (0%)		
60	1 (7.1%)	0 (0%)		
70	1 (7.1%)	0 (0%)		
90	1 (7.1%)	0 (0%)		
ESR, mean ± SD	46.57 ± 15.87	25.71 ± 12.53	0.018	T test
CRP, median (IQR)	9.32 (6.47, 46.8)	3.53 (2.01, 13.59)	0.097	Wilcoxon
CDAI, median (IQR)	22 (18.5, 28.5)	7 (2.5, 9.5)	0.002	Wilcoxon
SDAI, median (IQR)	22.93 (18.97, 34.95)	7.14 (2.76, 11.36)	0.002	Wilcoxon
DAS28-ESR, median (IQR)	5.5 (5.16, 5.88)	2.33 (2.27, 3.66)	< 0.001	Wilcoxon
DAS28-CRP, mean ± SD	5.16 ± 1.41	2.4 ± 1	0.001	T test
VAS, median (IQR)	5 (4.5, 5.5)	0.5 (0.5, 1.5)	0.004	Wilcoxon
HAQ, median (IQR)	0.5 (0.32, 3.56)	0 (0, 1.13)	0.195	Wilcoxon

According to different data types, different analysis methods are selected.

#### Diagnostic ROC analysis

In terms of predicting disease activity, CP (AUC=0.837), TTR (AUC=0.776), ORM1 (AUC=0.796), HP (AUC=0.816), SERPINA1 (AUC=0.796). They have a certain accuracy in the diagnosis and prediction of disease activity, but they are not statistically significant compared with other diagnosis results.

### Analysis of Differentially Expressed Proteins

**Figure 6:**
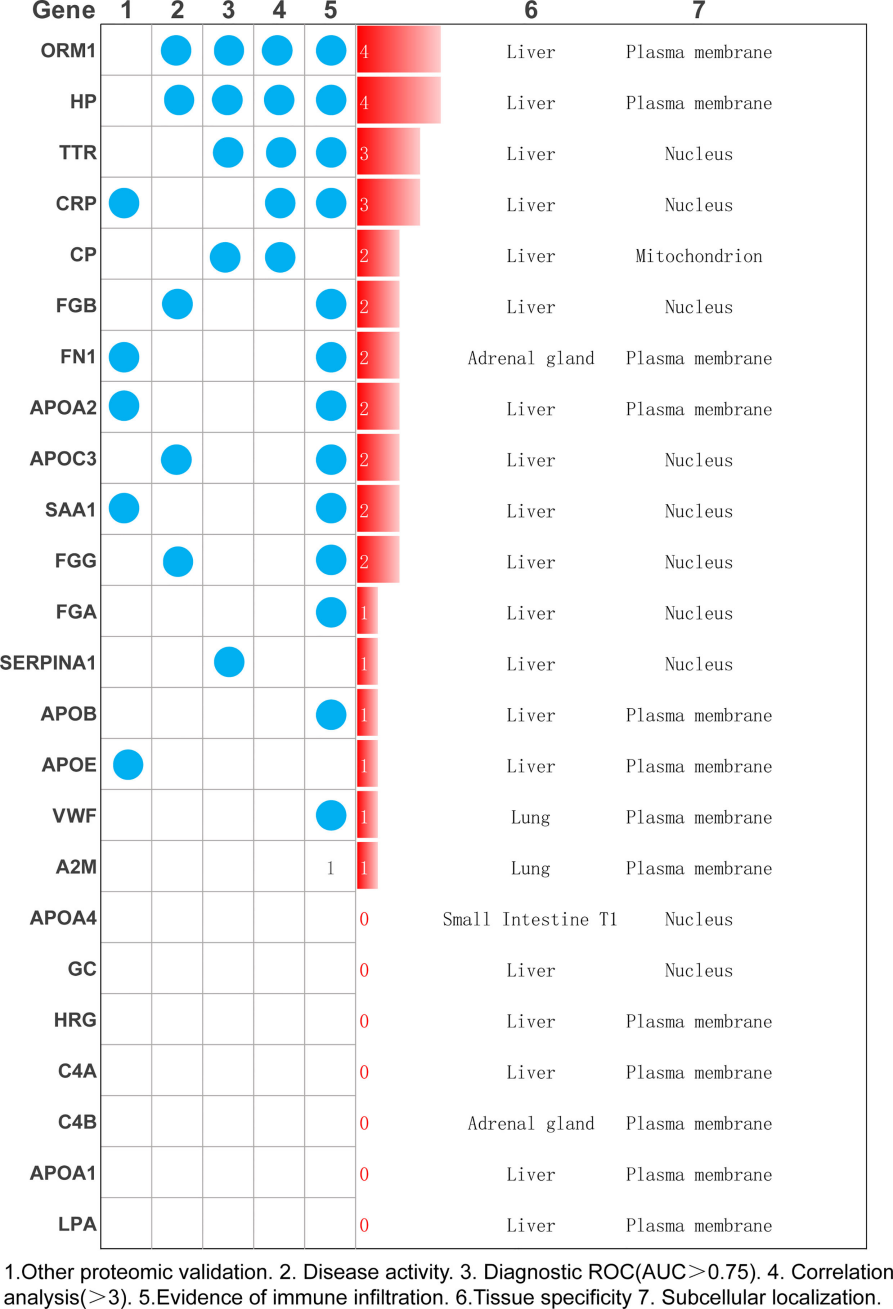
Summarize a variety of analysis methods to select the most meaningful protein as the key protein of RA.

As shown in **Figure 6**, we combined other proteomics verification conditions, immune infiltration conditions, disease activity, correlation analysis, and diagnostic ROC analysis to comprehensively analyze the 24 differential proteins. The results show that ORM1 and HP were differentially expressed and diagnosed in the disease activity and inactive phases. An ROC value greater than 0.7 has good diagnostic ability for the analysis of the active and inactive stages of the disease, is significantly related to more diagnostic indicators of RA, and has a good performance in terms of immune infiltration. ORM1 and HP show good distinguishing ability in the diagnosis and treatment of RA.

## Discussion

Through diverse-perspective analysis, our study provides novel insights into RA-related proteomics research. Using random forest machine learning methods and analysis of 11 other topological analysis methods, our proteomics data selected 24 potential differential proteins in normal and RA patient samples. When searching the PubMed database for 24 potential proteins, at least 19 (79.17%) were found in RA-related reports (**Table 3**). Meanwhile, 5 of the 24 differential protein species, including CRP, FN1, APOA2, SAA1, and APOE, have been verified in additional proteomics datasets. Separately, CRP, a diagnostic marker of RA in clinical practice, is significantly elevated in plasma levels in response to infection, inflammatory stimuli, or other tissue damage in the acute phase. Additionally, we found that our DEP list is significantly enriched in the other two DEP lists (**Table 1**) from proteomics in a gene background of 20,462 human protein-coding genes from the NCBI database, for example, dataset iv (*p* = 3.25 * 10^-32^, Fisher test) and dataset iii (*p* = 4.56 * 10^-31^, Fisher test). Collectively, we showed the reliability and accuracy of our protein list, and it is in accordance with previous publication. We will upload this information together. The above evidence demonstrates the reliability and accuracy of our analysis.

**Table 3:** Summary of human proteome datasets for biomarker analysis.

Official full name	Symbol	Total articles	RA-related articles	PMID	Level change
Orosomucoid 1	**ORM1**	3,555	1	26915672	Up
Haptoglobin	**HP**	10,621	80	4029960	Up
Transthyretin	**TTR**	10,864	13	30308029	Down
C-reactive protein	**CRP**	91,372	3,271	33385862	Up
Ceruloplasmin	**CP**	9,192	55	26001728	Up
Fibrinogen beta chain	**FGB**	1,453	6	26059223	Up
Fibronectin 1	**FN1**	24,248	2	34764682	Down
Apolipoprotein A2	**APOA2**	3,112	NA	NA	Down
Apolipoprotein C3	**APOC3**	734	NA	NA	Down
Serum amyloid A1	**SAA1**	444	2	17985847	Up
Fibrinogen gamma chain	**FGG**	441	NA	NA	Up
Fibrinogen alpha chain	**FGA**	2,059	NA	NA	Up
Serpin family A member 1	**SERPINA1**	4	2	34712268	Up
Apolipoprotein B	**APOB**	15,507	36	29997113	Down
Apolipoprotein E	**APOE**	27,281	15	32253242	Up
Von Willebrand factor	**VWF**	22,539	43	25973092	Up
Alpha-2-macroglobulin	**A2M**	5,061	43	10343526	Up
Apolipoprotein A4	**APOA4**	1,046	NA	NA	Up
GC vitamin D-binding protein	**GC**	849	1	3874814	Down
Histidine-rich glycoprotein	**HRG**	876	1	29246875	Up
Complement C4A	**C4A**	1,224	3	22076784	Down
Complement C4B	**C4B**	2,889	1	22076784	Up
Apolipoprotein A1	**APOA1**	14,603	15	31694752	Down
Lipoprotein A	**LPA**	9,068	37	19369465	Up

NA, not available.

FN1, a multidomain extracellular matrix (ECM) protein, binds to multiple compounds on the cell surface, including collagen (13), fibrin (14), growth factors (15), and cell surface integrins (16), and prominently participates in several fundamental biological processes, such as tissue repair (17), fibrosis (18), tumorigenesis (2016), cell adhesion (19), cell motility (20), and cell shape maintenance (21). Apolipoprotein A2 (APOA2) is the second most abundant protein of the high-density lipoprotein particles. The related pathway peroxisome proliferator-activated receptor (PPAR)-α regulates metabolism (22). SAA1 (serum amyloid A1) is a major acute-phase protein that is highly expressed in response to inflammation and tissue injury. Studies show that tumor necrosis factor α (TNF-α) and interleukin 1β (IL-1β) induce activation of the IL-6-STAT3 pathway to increase SAA1 expression (23). High levels of this protein are connected with chronic inflammatory diseases including rheumatoid arthritis (24), atherosclerosis (25), and Alzheimer’s disease (26). Apolipoprotein E (APOE) is a multifunctional cholesterol carrier that plays a central role in lipid metabolism in the peripheral and central nervous systems. Related pathways include the mTOR signaling pathway (27) and the AMPK signaling pathway (28), which are essential for the normal catabolism of triglyceride-rich lipoprotein components. These results not only verify the accuracy of our experiment but also provide a new direction for the research of RA fundamental research. At the same time, we found that lipid metabolism is closely related to the occurrence and development of RA according to our proteomics research and other proteomics verifications. Lipid levels are dynamic and can fluctuate along with changes in inflammation or as a result of disease-modifying antirheumatic drug (DMARD) therapy, which needs further study.

In addition, our research reevaluated the importance of 24 candidate proteins in terms of immune infiltration, disease activity, experimental diagnosis correlation analysis, and diagnostic ROC analysis. After a comprehensive analysis, both HP and ORM1 exceled in this aspect.

HP has many physiological functions. It can combine with free hemoglobin to prevent oxidative damage to various organs (29) and activate local and systemic levels of innate and adaptive immune cells, as an acute-phase reactant protein; plasma concentration greatly increases during inflammation (30). HP is mainly synthesized in the liver and rarely expressed in other inflamed tissues. It is synthesized at the injured site during acute-phase reactions such as inflammation and tissue damage. There are few reports about HP in RA, and it can be determined that the level in RA synovial fluid and arthritis tissue is related to disease activity (31). Previous studies have shown that serum haptoglobin levels increase during arthritis (32). Studies have shown that locally expressed HP promotes cell migration in cartilage and therefore may play a role in the progression of arthritis (31). Studies have determined that haptoglobin is an important factor in cell migration (33). Haptoglobin knockout cells exhibit impaired migration, which can be restored by supplementing the cells with exogenous haptoglobin. Our research once again verified the importance of HP and confirmed that the immunomodulatory effect of HP is related to CD56dim natural killer cells, effector memory CD8^+^T cells, and natural killer cells.

Orosomucoid 1 (ORM1) is different from HP in that it has less research. When we searched in PubMed, there were only 3,555 related articles (**Table 3**), which is more meaningful and valuable from the perspective of innovation. ORM1 is a key acute-phase plasma protein, which is related to acute inflammation. Its specific function has not yet been determined, which may involve immunosuppressive aspects. ORM1 is rich in liver tissue, secreted into the blood. Subcellular locations of ORM1 are mainly in the extracellular space; the plasma membrane is more abundant than the mitochondrion and nucleus. The immunomodulatory effect of ORM1 is related to CD56dim natural killer cells, effector memory CD8^+^T cells, and natural killer cells. ORM has many activities including, but not limited to, acting as an acute-phase reactant and disease marker, modulating immunity, binding and carrying drugs, maintaining the barrier function of the capillary, and mediating the sphingolipid metabolism (34). In RA patients, a study of the combination of transcriptome and proteome in human urine showed that urinary ORM1 levels in RA patients had a positive correlation with the status of the disease activity (35). Although the literature in RA is limited, ORM has been shown to have anti-inflammatory protective effects in other diseases. Exogenous ORM can significantly reduce the infarct size and neurological deficit score of ischemic stroke. The specific mechanism is to inhibit the production of IL-1β, IL-6, and TNF-α, significantly reduce inflammation, improve malondialdehyde (MDA) and superoxide dismutase (SOD) balance to reduce oxidative stress, and reduce the activity of caspase-3 to inhibit apoptosis (36). Based on data obtained from the Gene Expression Omnibus (GEO) database, previous research revealed that ORM1 was highly expressed and positively correlated with the expression of inflammatory factors (MAPK1, MAPK3, IL1B, and CASP9) (37). In general, ORM1 is a protein related to disease activity and is expected to become a marker for the treatment and diagnosis of RA. Our research group will study in depth the specific mechanism of ORM1 in future research.

However, one of the disadvantages in this experiment design is that proteins are the performers of functions; it is necessary to consider combining the results of transcriptome and metabolomics for multidimensional research. Also, the serum proteome has its limitations, and the perspective of synovial proteomics is worth further studying. If possible, the systematic research on the differentially expressed proteins should be screened out, combined with animal experiments, clinical trials, etc., using molecular biology techniques to verify and enrich the rationality of high-throughput screening results.

The innovation of this study is that we refer to the results of other RA proteomic studies to make our findings more convincing. Our analysis of differential proteins, combined with disease activity, is more clinically meaningful and valuable. In general, these rich data are expected to stimulate subsequent hypothesis-driven research, deepen our understanding of the pathogenesis of RA, provide potential biomarkers, and provide new strategies to facilitate the diagnosis and treatment of this harmful disease.

## Data Availability Statement

The data presented in the study are deposited in the PRIDE and ProteomeXchange repository. ProteomeXchange accession number PXD032912.

## Ethics Statement

The studies involving human participants were reviewed and approved by the First Affiliated Hospital of Guangzhou University of Chinese Medicine Ethics Committee.[2020]264. The patients/participants provided their written informed consent to participate in this study. Written informed consent was obtained from the individual(s) for the publication of any potentially identifiable images or data included in this article.

## Author Contributions

GC, LW, and CH conceived and designed the experiments. ZD and JX conducted the experiments and wrote the manuscript. LiZ, YX, ML, JY, LuZ, and HD provided some of the data. LL, MZ, JH, LW, and GC reviewed and revised the manuscript. CH and ZD have contributed equally to this work. All authors contributed to the article and approved the submitted version.

## Funding

This study was funded by the Guangzhou Science Technology and Innovation Commission Technology Research Projects (No. 201904010336) and the Innovative and Strong Hospital Project of The First Affiliated Hospital of Guangzhou University of Chinese Medicine (No. 211010010705).

## Conflict of Interest

The authors declare that the research was conducted in the absence of any commercial or financial relationships that could be construed as a potential conflict of interest.

## Publisher’s Note

All claims expressed in this article are solely those of the authors and do not necessarily represent those of their affiliated organizations, or those of the publisher, the editors and the reviewers. Any product that may be evaluated in this article, or claim that may be made by its manufacturer, is not guaranteed or endorsed by the publisher.
